# Effect of oral rehydration solution versus spring water intake during exercise in the heat on muscle cramp susceptibility of young men

**DOI:** 10.1186/s12970-021-00414-8

**Published:** 2021-03-15

**Authors:** Wing Yin Lau, Haruyasu Kato, Kazunori Nosaka

**Affiliations:** 1 Center for Exercise and Sports Science Research, School of Medical and Health Sciences, Edith Cowan University, 270 Joondalup Drive, Joondalup, WA 6027 Australia; 2 Department of Sport and Wellness, Rikkyo University, Niiza, Saitama Japan

**Keywords:** Electrical train stimulation, Threshold frequency, Downhill running, Sodium, Chloride, Oral rehydration solution, Hyponatremia

## Abstract

**Background:**

Muscle cramp is a painful, involuntary muscle contraction, and that occurs during or following exercise is referred to as exercise-associated muscle cramp (EAMC). The causes of EAMC are likely to be multifactorial, but dehydration and electrolytes deficits are considered to be factors. This study tested the hypothesis that post-exercise muscle cramp susceptibility would be increased with spring water ingestion, but reduced with oral rehydration solution (ORS) ingestion during exercise.

**Methods:**

Ten men performed downhill running (DHR) in the heat (35–36 °C*)* for 40–60 min to reduce 1.5–2% of their body mass in two conditions (spring water vs ORS) in a cross-over design. The body mass was measured at 20 min and every 10 min thereafter during DHR, and 30 min post-DHR. The participants ingested either spring water or ORS for the body mass loss in each period. The two conditions were counter-balanced among the participants and separated by a week. Calf muscle cramp susceptibility was assessed by a threshold frequency (TF) of an electrical train stimulation to induce cramp before, immediately after, 30 and 65 min post-DHR. Blood samples were taken before, immediately after and 65 min after DHR to measure serum sodium, potassium, magnesium and chroride concentrations, hematocrit (Hct), hemoglobin (Hb), and serum osmolarity. Changes in these varaibles over time were compared between conditions by two-way repeated measures of analysis of variance.

**Results:**

The average (±SD) baseline TF (25.6 ± 0.7 Hz) was the same between conditions. TF decreased 3.8 ± 2.7 to 4.5 ± 1.7 Hz from the baseline value immediately to 65 min post-DHR for the spring water condition, but increased 6.5 ± 4.9 to 13.6 ± 6.0 Hz in the same time period for the ORS condition (*P* < 0.05). Hct and Hb did not change significantly (*P* > 0.05) for both conditions, but osmolarity decreased (*P* < 0.05) only for the spring water condition. Serum sodium and chloride concentrations decreased (< 2%) at immediately post-DHR for the spring water condition only (*P* < 0.05).

**Conclusions:**

These results suggest that ORS intake during exercise decreased muscle cramp susceptibility. It was concluded that ingesting ORS appeared to be effective for preventing EAMC.

## Background

Water is essential for our body, thus it is necessary to euhydrate by drinking fluids and eating foods that contain water every day. Exercising in the heat increases body and core temperature, and reduces water content in the body, therefore fluid intake is crucial for maintaining performance and preventing dehydration that could cause serious events [1, 2]. However, drinking too much plain water during exercise may negatively affect exercise performance [3, 4]. Rosner and Kirven [5] stated that one of the risk factors for “exercise-associated hyponatremia” was excessive drinking (1.5 L/h during the event). Hoffman and Stuempfle [6] demonstrated that overhydration was the primary characteristic of symptomatic hyponatremia during a 161-km running race. One of the symptoms of hyponatremia is muscle cramping, which is a painful, involuntary muscle contraction [7, 8]. When muscle cramping occurs during and/or following exercise, it is referred to as exercise-associated muscle cramp (EAMC) [9–11]. It is possible that drinking a large amount of plain water dilutes sodium and other electrolytes in the blood and extracellular fluid, increasing EAMC susceptibility [12–14].

It has been reported that the prevalence of EAMC among participants is 39% in marathons, 52% in rugby, 60% in cycling, and 68% in triathlons [15]. Schwellnus et al. [16] reported that 20% of triathletes experienced muscle cramp either once or multiple times during and/or within 6-h after an Ironman triathlon race. The mechanisms underpinning EAMC are unknown, but are likely to be multifactorial [10, 11, 15, 17]. Giuriato et al. [18] have reported that EAMC stems from an imbalance between excitatory drive from muscle spindles and inhibitory drive from Golgi tendon organs to the alpha motor neurons, rather than dehydration or electrolytes deficits. On the other hand, Maughan and Shirreffs [7] have stated in their recent review paper that high ambient temperature and large sweat losses accompanied by the ingestion of large volumes of plain water may be risk factors for EAMC. Therefore, it is interesting to examine the effects of plain water versus water containing electrolytes on EAMC to clarify whether any differences exist between the conditions.

To quantify muscle cramp susceptibility, previous studies [14, 19–23] used electrical stimulation to induce muscle cramp, and showed that muscle cramp was induced by increasing the electrical stilumation frequency, and that threshold frequency which induced muscle cramp could be used as an indicator of muscle cramp susceptibility. For example, Lau et al. [14] reported that spring water ingestion after dehydration equivalent to 2% of body mass induced by downhill running in the heat (35–36 °C), increased muscle cramp susceptibility assessed by a threshold frequency (TF) of electrical train stimulation to induce cramp. However, when oral rehydration solution (ORS: please refer to the contents of OS-1 shown below) was ingested after exercise, TF increased, indicating decreased muscle cramp susceptibility. These results suggested that plain water intake after dehydration made muscles more susceptible to muscle cramp, but when ORS was consumed, muscle cramp susceptibility was reduced [14]. However, it is not known how plain water or ORS ingestion during exercise in the heat affects muscle cramp susceptibility.

Therefore, the present study compared changes in TF of calf muscles before and after running in the heat (35–36 °C) with the two conditions of spring water versus ORS intake during exercise for the amount of body mass loss by sweating every 10–20 min. Since no changes in TF were observed by dehydration itself (loss of 1% or 2% of body mass), and TF decreased after spring water intake in the previous study [14], it was hypothesized that TF would be decreased (muscle cramp would be induced by a lower frequency of electrical train stimulation) when spring water was ingested, but increased when ORS was ingested during the running.

## Methods

### Participants

This study was approved by the Institutional Human Research Ethics Committee, and complied with the Declaration of Helsinki. Ten young men were recruited for the present study, and each of them signed an informed consent form and completed medical questionnaires before participating in the study. The effect size for the difference in TF changes between conditions was estimated to be 0.9 based on our previous study [14], and 10 participants were shown to be adequate with the alpha level of 0.05 and power (1 − β) of 0.80. Their mean ± SD (range) age, height and body mass were 25.0 ± 2.7 (22–31) years, 173.7 ± 6.4 (165–184) cm and 74.0 ± 12.0 (57.2–89.3) kg, respectively.

All participants were in good health and fitness, participated in moderate exercise and sporting activities 2–3 times a week (less than 300 min in total), and were not prone to muscle cramping. However, participants responded to the electrical train stimulation described below and had muscle cramping in the screening. This was checked in a familiarization session that was set 7–10 days before the first experimentas session. If participants did not tolerate to the electrical stimulation, or no muscle cramping was induced by the electrical stimulation, they were excluded from the study. Thus, all participants included in the present study were muscle cramp responders to the electrical stimulation. In the familiarizarion session, the participants also experienced downhill running (DHR) with the slope of 5% for 10 min at 5.5–6.0 km·/h^− 1^. They were not exposed to temperature higher than 32 °C during the 4 weeks prior to this study.

### Study design

The present study used OS-1 (Otsuka Pharmaceutical Factory, Inc., Japan) containing sodium (1150 mg/L = 50 mM/L), potassium (780 mg/L = 20 mM/L), magnesium (24 mg/L = 1 mM/L), chloride (1770 mg/L = 50 mM/L), glucose (18,000 mg/L = 100 mM/L) and others (e.g., phosphorus) as ORS. For the other condition, spring water (Coles Natural Spring Water, Coles, Australia) was used which contained a small amount of sodium (2 mg/L), potassium (0.5 mg/L), magnesium (18 mg/L), chloride (1.2 mg/L), and calcium (39 mg/L). The fluid ingested during and after DHR was the same for each condition. Using a cross-over design, the OS-1 and spring water conditions were compared for changes in TF of electrical stimulation to induce calf muscle cramp before and after DHR. The two conditions were counterbalanced among the participants and separated by a week. DHR was used in the present study, since its metabolic demand is smaller than that in level or uphill running [24]. Thus, DHR was easier for the particiopants to perform, but induced relatively large sweating of nearly 2% of body mass in less than 60 min [14].

### Muscle cramp assessment

To assess calf muscle cramp susceptibility, calf muscles were electrically stimulated to induce muscle cramping, and the frequency of the stimulation to induce muscle cramp was used as an indicator of muscle cramp susceptibility [14]. Each participant lay prone on a massage bed, and the instep was placed on the bed, which kept the ankle joint in a plantar-flexed position. Electrical train stimulation was delivered to the calf muscles of the kicking (dominant) leg by a portable electrical stimulator (Compex 2, Compex Medical, Switzerland) with one electrode (cathode) placed over the tibialis posterior nerve in the popliteal fossa, and the other electrode (anode) placed at the tibialis tendon. The locations of the electrodes were marked by a semi-permanent marker to ensure the consistent electrode placement between measures on the same day and between sessions separated by a week. Each stimulation consisted of 0.5-s duration of rise time and 2-s bursts of stimuli of 300-μs duration, which was specifically programmed for the present study. The stimulation started at a frequency of 10-Hz, and two stimulations were given at this frequency during which the stimulation intensity was increased to a level (18–60 mA) which had been determined in a familiarisation session. The intensity of the stimulation was set for each participant to have muscle cramp at 24 or 26 Hz, and the same intensity was used in all measurements. This method was developed for the present study, based on our previous study [14]. The intensity (amplitude) of the stimulation varied among participants (40–60 mA), but all of them had muscle cramping at 24 or 26 Hz at the baseline. The stimulation frequency was automatically increased by 2 Hz from 10 Hz every 30 s until muscle cramp was induced, and the TF at which cramp was induced was recorded. The muscle cramp was identified by a visibly taut muscle sustained after stimulation, and pain reported by the participant. Participants were instructed to relax during the electrical stimulation, and as soon as muscle cramp was confirmed, the cramp was relieved by passive dorsiflexion of the foot by the investigator.

### Exercise

All participants were instructed to refrain from any strenuous exercise for one week prior to participating in the study. They were asked to consume 600-ml of spring water at 2 h before coming to the laboratory, and refrain from any food and beverage intake thereafter. All participants were required to record their food intake before the first session, and they were asked to have the same foods and amount of water before the second session. However, the actual food and fluid intakes were not checked nor recorded, thus it was not not known whether the meal content and fluid intake before the two sessionas were identical.

**Fig. 1:**
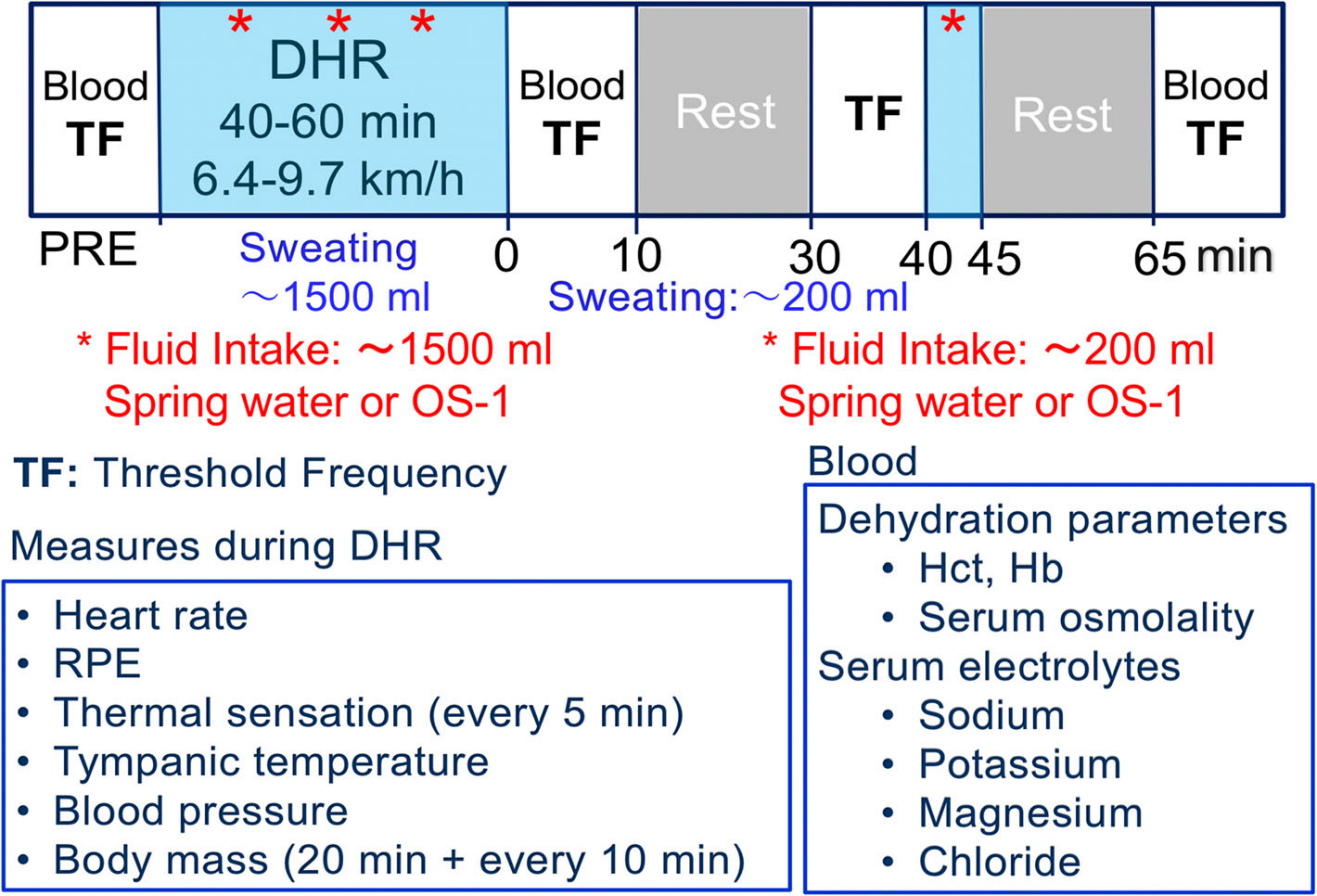
Study design and the time course of measurements taken in the study. Before downhill running (DHR), blood sample was taken to assess haematocrit (Hct), haemoglobin and serum osmolality, and to measure serum sodium, potassium, magnesium and chloride concentrations, and threshold frequency (TF) of electrical train stimulation to induce muscle cramp was measured as an indicator of muscle cramp susceptibility. During DHR, heart rate were monitored continuously, rate of perceived exertion (RPE) and thermal sensation were recorded every 5 min, and tympanic temperature, blood pressure and body mass were measured after the first 20 min followed by every 10 min during DHR. The participants ingested spring water or OS-1 (indicated by *) for the volume equivalent to the body mass loss by sweat for the first 20 min and every 10 min thereafter for the pre-determined exercise duration (40–60 min) after each body mass measure (approximately 1500 ml in total). Immediately after DHR, blood sample was taken for the analyses shown above, and TF was measured again. TF was measured at 30 and 65 min after DHR, and blood sample was taken immediately before the TF measures at 65 min post-DHR. Spring water or OS-1 was ingested at 40 min post-exercise (indicated by *) for the lost amount of body mass between immediately post- to 40 min post-exercise (approximately 200 ml)

The participants performed two bouts of DHR (slope: 5%) in a climate chamber at 35–36 °C and 25–28% relative humidity (Fig. 1). The running intensity and duration to reduce 2% of body mass (1.14–1.78 L) without fluid intake were based on the previous study [14]. The running velocity was between 6.4–9.7 km/h among the participants, and the velocity was modified for each participant. The body mass was measured by a scale (Mettler Toltdo ID1, Columbus, OH, USA) after the first 20 min of DHR, when each participant stopped running, took off all clothes and shoes, and wiped off sweat. This was repeated every 10 min thereafter for the same duration as that of the previous study (40–60 min) [14]. After the body mass measurement, each participant ingested either spring water or OS-1 for the amount of the body mass decrease in the time period (Fig. 1). In the second bout, the protocol was the same as that of the first bout, thus the participants ran the same duration for the same distance at the same velocities for the two bouts.

During DHR, heart rate (HR) (Model S610i; Polar Electro Oy, Finland) and rating of perceived exertion (RPE, 6–20 point Borg Scale) were recorded (Fig. 1). Ratings of perceived thermal sensation was assessed with an 8-point thermal rating scale (0: unbearably cold to 8: unbearably hot) [25]. These were measured before DHR, then every 5 min during DHR. Blood pressure and tympanic temperature were measured by an automatic sphygmomanometer and a digital ear thermometer (BraunThermoScan 5, USA), respectively, before, after the first 20 min and every 10 min during DHR, and at the end of DHR, when each participant stopped running and was sitting in a chair.

### Blood analyses

Approximately 8 ml of blood was drawn by a standard venepuncture from the antecubital vein before, immediately after, and 65 min after DHR (Fig. 1), while each participant was sitting on a phlebotomy chair. A portion of the blood sample (1.5 ml) was used to measure hematocrit (Hct) and hemoglobin (Hb) by a capillary method and a HemoCue (Hb 201 System, Sweden), respectively, and the plasma volume change was calculated [26]. The normal reference range was 14–18 g/dl for Hb, and 40–54% for Hct in young adults [27]. The rest of the blood was centrifuged for 10 min at 3000 rpm to obtain serum for the analyses of electrolyte concentrations of sodium, chlorine, potassium and magnesium, and osmolality. The electrolyte concentrations were measured by an ABBOTT Architect C160000 analyser (Abbott Park, IL, USA) using a corresponding kit for sodium, chlorine and potassium and using another kit for magnesium (Abbott Laboratories Diagnostics, Abbott Park, IL, USA). Normal reference range of serum concentration for young adults by the method was sodium: 136–146 mmol/L, potassium: 3.5–4.5 mmol/L, magnesium: 0.65–1.05 mmol/L and chlorine: 96–106 mmol/L.

### Statistical analyses

Data were assessed by a Shapiro-Wilk test for the normality and a Levene test for the homogeneity of variance assumption. Two-way repeated measures of analysis of variance (ANOVA) was used to compare between conditions (spring water vs OS-1) for the changes in TF before, immediately after, and 30 and 65 min post-DHR, changes in body mass, HR, RPE, thermal sensation, blood pressure, and tympanic temperature during DHR, and changes in Hct, Hb, serum osmolality, and serum electrolyte concentrations (sodium, potassium, magnesium, chloride) before, immediately after and 65 min after DHR. When the ANOVA showed a significant time effect and/or interaction effect, a Tukey’s post-hoc test was performed for multiple comparisons. Correlations between the changes in TF and serum electrolyte concentrations were assessed by a Pearson’s product-moment. Statistical significance was set at *P* < 0.05, and all data were presented as mean ± standard deviation (SD).

## Results

### Exercise

The distance covered in the DHR was 5.6–9.0 km among the participants, and the average velocity was 8.0 ± 1.2 km/h among the participants, which were the same between conditions. The variability among the participants was due to the velocity used in the DHR depending on the fitness level of the participants. The average HR, RPE and thermal sensation during DHR were 140.3 ± 17.9 bpm, 11.6 ± 2.2, and 5.2 ± 0.6, respectively, without a significant difference between conditions. Systolic blood pressure increased and diastolic blood pressure decreased by approximately 12 mmHg and 14 mmHg, respectively from the baseline during DHR. The tympanic temperature increased from 36.4 ± 0.2 °C before DHR to 37.5 ± 0.3 °C at the end of DHR for both conditions. No particpants experirnced muscle soreness after DHR.

The total amount of fluid intake during DHR was 1223.0 ± 241.8 ml (range: 918–1741 ml) for the spring water and 1294.0 ± 299.6 ml (range: 903–1848 ml) for the OS-1 condition, without a significant difference between conditions. In 30 min after DHR, body mass decreased approximately 200 g for both conditions, and this amount was replenished by spring water (198.3 ± 143.0 ml) or OS-1 (215.0 ± 140.3 ml). The total amount of fluid intake during and after exercise was not significantly different between conditions (1421.0 ± 314.6 ml vs 1509.0 ± 392.0 ml).

No significant differences between conditions were found for the baseline values of Hct, Hb and serum osmolality (Table 1). Hct and Hb did not change significantly immediately after DHR for both conditions, but serum osmolarity decreased immediately post-DHR for the spring water condition only. Percent changes in plasma volume were calculated using the equation by [28]. Plasma volume increased 6.2% from baseline to immediately post-exercise for the OS-1 condition, but only 1.6% for the spring water condition. No muscle cramp occured during DHR in both conditions.

**Table 1:** Changes (mean ± SD, range, *95% confidence interval*) in haematocrit (Hct), haemoglobin (Hb) and osmolality, serum concentration of sodium, potassium magnesium and chloride before (Pre), and immediately (0) and 65 min following downhill running (DHR) for the spring water (Water) and electrolyte water (OS-1) intake during DHR and 40 min after DHR. *: significant (*P* < 0.05) difference from the pre-value, #: significant (*P* < 0.05) difference between conditions

	Condition	Pre	0	65	ANOVA
**Hct** **(%)**	Water	45.7 ± 2.5	45.2 ± 2.5	45.5 ± 2.5	F = 2.92 *P* = 0.08
		(42.0–48.3)	(41.3–48.0)	(41.3–48.0)	
		*44.2–47.2*	*43.7–46.7*	*43.9–47.1*	
	OS-1	47.6 ± 4.0	45.7 ± 1.8	45.3 ± 1.5*	
		(42.6–50.0)	(42.6–48.3)	(43.3–48.0)	
		*45.1–50.1*	*44.6–46.8*	*44.4–46.3*	
**Hb** **(g/dl)**	Water	15.5 ± 1.2	15.4 ± 1.3	15.4 ± 1.2	F = 1.51 *P* = 0.24
		(13.8–16.9)	(13.7–17.3)	(13.7–16.9)	
		*14.8–16.2*	*14.6–16.2*	*14.6–16.1*	
	OS-1	15.6 ± 1.1	15.2 ± 0.9	15.1 ± 0.9*	
		(14.0–17.0)	(14.2–16.5)	(13.5–16.4)	
		*14.8–16.3*	*14.6–15.8*	*14.5–15.8*	
**Osmolality** **(mOsm/kg H**_**2**_**O)**	Water	291.1 ± 4.9	285.3 ± 6.8*	288.6 ± 6.1	F = 5.27 *P* = 0.016#
		(282–300)	(277–300)	(281–301)	
		*288.0–294.1*	*281.1–289.5*	*284.8–292.4*	
	OS-1	293.3 ± 6.2	294.2 ± 7.5	289.7 ± 3.8*	
		(282–301)	(285–307)	(285–296)	
		*289.5–297.1*	*289.5–298.9*	*287.3–292.1*	
**Sodium** **(mmol/L)**	Water	140.2 ± 1.9	137.9 ± 2.2*	138.8 ± 1.9	F = 0.92 *P* = 0.42
		(137–143)	(135–142)	(137–140)	
		*139.0–141.3*	*136.6–139.3*	*137.6–140.0*	
	OS-1	140.7 ± 2.9	139.3 ± 4.6	139.1 ± 2.3*	
		(136–145)	(138–146)	(136–144)	
		*138.9–142.5*	*136.5–142.1*	*137.6–140.6*	
**Potassium** **(mmol/L)**	Water	4.6 ± 0.4	4.5 ± 0.4	4.2 ± 0.5*	F = 1.0 *P* = 3.87
		(4.0–5.3)	(4.1–5.1)	(3.5–4.9)	
		*4.3–4.8*	*4.2–4.8*	*3.8–4.5*	
	OS-1	4.7 ± 0.6	4.7 ± 0.6	4.4 ± 0.6*	
		(4.1–5.8)	(4.0–5.9)	(3.8–5.4)	
		*4.3–5.1*	*4.3–5.1*	*4.0–4.8*	
**Magnesium** **(mmol/L)**	Water	0.85 ± 0.04	0.82 ± 0.05*	0.84 ± 0.06	F = 1.85 *P* = 0.19
		(0.78–0.91)	(0.71–0.89)	(0.70–0.94)	
		*0.83–0.87*	*0.78–0.85*	*0.80–0.88*	
	OS-1	0.89 ± 0.04	0.83 ± 0.04*	0.86 ± 0.05*	
		(0.83–0.96)	(0.77–0.92)	(0.78–0.93)	
		*0.86–0.92*	*0.81–0.86*	*0.83–0.90*	
**Chloride** **(mmol/L)**	Water	101.9 ± 2.0	100.0 ± 2.4*	100.3 ± 2.6	F = 16.8 *P* = 0.001#
		(99–105)	(96–104)	(97–104)	
		*100.6–103.2*	*98.5–101.5*	*98.7–101.9*	
	OS-1	101.6 ± 2.7	102.7 ± 2.7	102.4 ± 2.0	
		(99–106)	(99–107)	(100–106)	
		*99.9–103.3*	*101.0–104.4*	*101.2–103.6*	

### Muscle cramp susceptibility

**Fig. 2:**
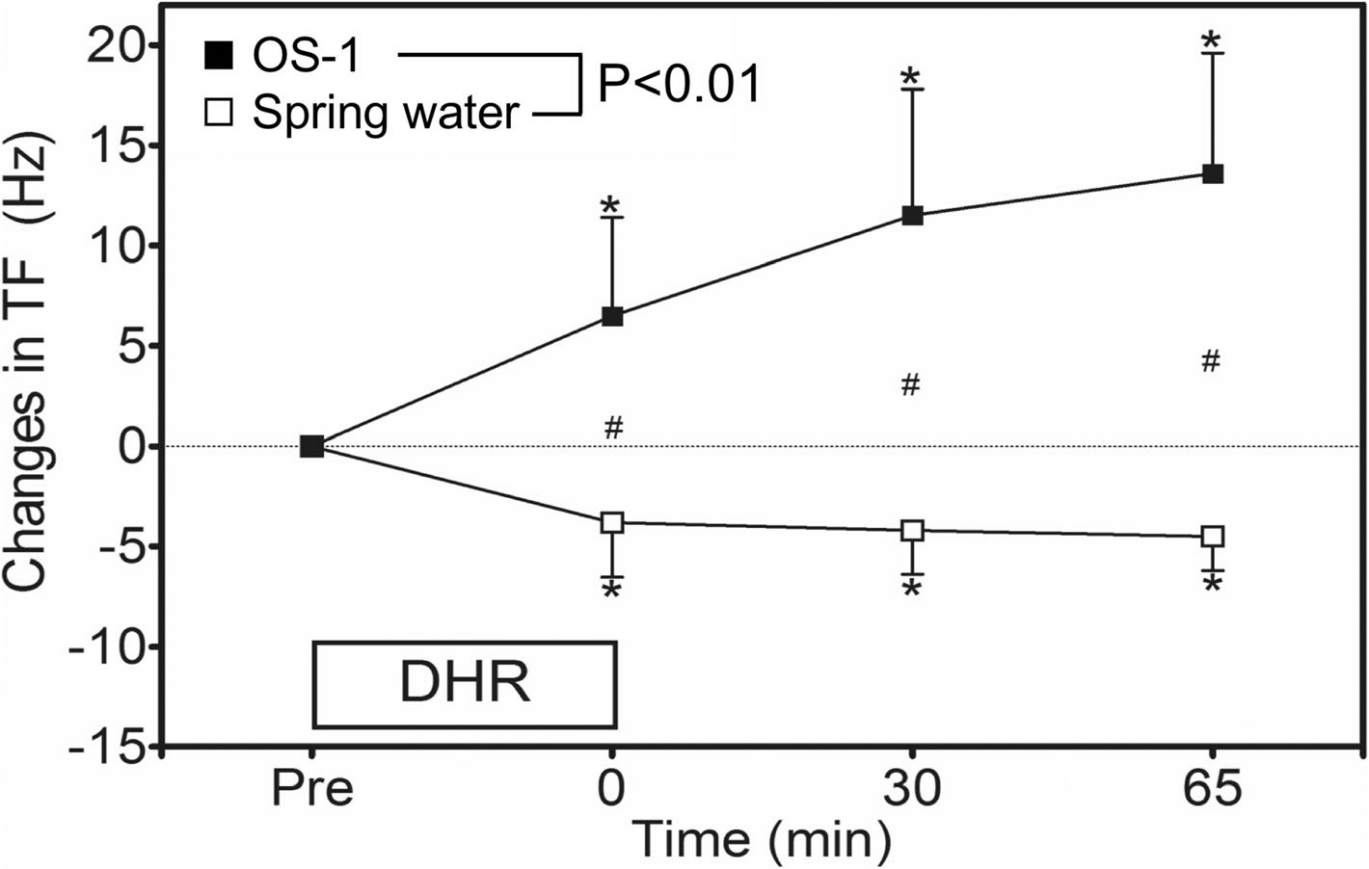
Absolute changes in threshold frequency (TF) of electrical train simulation to induce muscle cramp from the baseline (pre), at immediately after (0), 30 and 65 min after downhill running (DHR) in the heat for the spring water and OS-1 intake conditions. A significant (*P* < 0.01) interaction effect was found. * indicates a significant (*P* < 0.05) difference from the baseline (pre) value. # indicates a significant (*P* < 0.05) difference between the conditions

TF at baseline was not significantly different between the spring water (25.8 ± 0.6 Hz, 95% CI: 25.4–26.2 Hz) and OS-1 (25.6 ± 0.8 Hz, 25.1–26.1 Hz) conditions, but changes in TF (absolute values) were significantly (*P* < 0.01) different between conditions. Figure 2 shows absolute changes in TF following DHR from the baseline. TF decreased by 3.8 ± 2.7 Hz (95% CI: 2.1–5.5 Hz), 4.2 ± 2.2 Hz (2.8–5.6 Hz) and 4.5 ± 1.7 Hz (3.4–5.6 Hz) at immediately, 30 min and 65 min post-DHR, respectively from the baseline for the spring water condition. In contrast, TF increased by 6.5 ± 4.9 Hz (3.4–9.6 Hz), 11.5 ± 6.4 Hz (7.5–15.5 Hz) and 13.6 ± 6.0 Hz (9.8–17.3 Hz) for the respective time points for the OS-1 condition.

### Serum electrolytes

As shown in Table 1, no significant differences in serum electrolyte concentrations were evident between conditions at the baseline. No significant differences were found between conditions for the changes in all electrolytes except chloride, but significant decreases in serum sodium and chloride concentrations were observed only for the spring water condition at immediately post-exercise. No significant correlations were evident between the magnitude of change in TF and the magnitude of changes in serum sodium (r = 0.12, 95% CI: − 0.55–0.70) or chloride concentration (r = 0.24, − 0.46–0.75) at immediately post-exercise for the spring water condition.

## Discussion

The results of the present study showed that the TF to induce muscle cramp after DHR increased with the ingestion of OS-1 during DHR, but decreased with spring water ingestion during DHR. This supported the hypothesis that post-exercise muscle cramp susceptibility would be decreased by ingestinon of ORS but increased by spring water during exercise.

In comparison to the previous study [14], in which no fluid intake was allowed during DHR, the increases in HR (159.1 ± 3.0 bpm vs 140.3 ± 17.9 bpm), RPE (13.2 ± 2.0 vs 11.6 ± 2.2), thermal sensation (5.9 ± 0.9 vs 5.2 ± 0.6), and tympanic temperature (38.7 ± 0.6 °C vs 37.5 ± 0.3 °C) were smaller in the present study. However, the total amount of loss of body mass by sweat in the present study (1258 ± 267 g) was similar to that of the previous study (1304 ± 300 g) [14]. It appears that the fluid intake during DHR reduced the increase in core temperature, which lowered HR and RPE. As shown in Table 1, no significant changes in Hct and Hb from baseline to immediately after DHR were observed for both conditions. Furthermore, plasma volume was greater for the OS-1 than spring water condition from the baseline to immediately post-exercise, suggesting that water absorption was greater for the OS-1 condition. Serum osmolality and sodium concentration slightly decreased for the spring water condition, suggesting that sodium concentration in the blood was more diluted after the spring water than OS-1 ingestion. However, it is important to note that serum sodium concentration at immediately post-DHR did not reach the level of hyponatremia (< 135 mmol/L) for both conditions. This may be due to the relatively shorter duration and lower-intensity of the exercise. It does not appear that a decrease in serum sodium concentration per se is a cause of muscle cramp. However, it is possible that sodium or other electrylyte concentrations of inside and outside of muscle fibers are changed without changes in their serum concentrations. It is interesting to use a microdialysis or other technique to investigate changes in intracellular and extracellular electrolytes after spring water versus OS-1 or other ORS.

It should be noted that TF measures do not provide cramping intensity and duration, nor the extent of pain associated with the muscle cramp. However, TF has been used to assess muscle cramp susceptibility in the previous studies for the abductor halluces muscle [22, 23, 29] or the plantar flexors [14, 19]. Minetto et al. [22] were the first to use TF to examine muscle cramp, and demonstrated good inter-session (ICC = 0.82–0.92) and inter-day (ICC = 0.85) reliability results, and stated that TF was a reliable tool to assess a change in muscle cramp susceptability. Behringer et al. [19] showed that neuromuscular electrical stimulation performed twice a week for 6 weeks reduced the number of spontaneous calf cramps by 78%, and this was accompanied by an increase in the cramp threshold frequency from 15.5 ± 8.5 Hz to 21.7 ± 12.4 Hz. In the present study, the baseline TF was consistent (25.8 ± 0.6 Hz) in the two baseline measures with a week apart, indicating good test-retest reliability. Although the present study was not able to assess muscle cramp susceptibility during DHR exercise, the decrease in TF at immediately post-DHR is likely to indicate increased muscle cramp susceptibility during exercise. The average magnitude of decrease in TF in the present study for the spring water condition was around 4 Hz (Fig. 2). This finding was in line with the study by Miller et al. [28] who reported that a decreased threshold frequency to induce muscle cramp by 4 Hz indicated an increase in muscle cramp susceptibility. Thus, the decrease (4 Hz) is considered to be physiologically significant, and a change in TF was likely to indicate a change in muscle cramp susceptibility.

In regards to the relationship between dehydration (serum sodium concentration) and muscle cramp, Schwellnus et al. [29] reported that serum sodium concentration immediately after a 56-km road race was significantly lower for the cramping group (139.8 ± 3.1 mmol/L) than the non-cramping group (142.3 ± 2.1 mmol/L). Sulzer et al. [30] found a significant difference in serum sodium concentration after Ironman triathlons between the cramping (140 ± 2 mmol/L) and non-cramping (143 ± 3 mmol/L) groups, but they did not consider that this was clinically significant. In contrast, Hoffman and Stuempfle [6] showed no difference in serum sodium concentration at the finish of a 161-km ultramarathon among those with muscle cramping, near cramping and no cramping in the last stage of the race. Using TF to assess muscle cramp, Miller et al. [21] did not find changes in TF of electrically induced muscle cramp after 3% dehydration, in which an increase in serum sodium concentration from baseline (138.6 ± 0.2 mmol/L) to post-dehydration (145.1 ± 0.5 mmol/L) was found. Braulick et al. [20] reported an increase in serum sodium concentration in 3–5% dehydrated condition (149.5 ± 1.8 mmol/L) in comparison to euhydrated condition (141.9 ± 3.1 mmol/L), and found no difference in TF between the conditions. In the present study, the differences in serum sodium and chloride concentrations between the conditions were small at immediately after and 65 min post-DHR (Table 1), but TF was largely different between the conditions (Fig. 2). Additionally, no significant correlations were evident between the magnitude of change in TF and the magnitude of changes in serum sodium or chloride concentration. These suggest that muscle cramp susceptibility is not determined by serum sodium and chloride concentrations alone.

One of the limitations of the present study was that a control condition of no fluid ingestion during exercise was included to compare with other conditions. However, our previous study [14] showed that TF did not change significantly at immediately after DHR from the baseline without fluid intake during exercise. Thus, it seems likely that no significant change in TF would have been observed, if no fluid ingestion during exercise condition had been included. It is possible that an individual’s experiences in running, intensity (velocity) of the downhill running, and the order of the conditions affected the results. However, the slope of the DHR was not steep, and none of the participants experienced any delayed onset muscle soreness after the first bout. Moreover, the order of the two conditions was counterbalanced among the participants, and it appeared that all participants recovered fully from the previous bout in 1 week. Thus, it seems likely that the results reflected the fluid conditions rather than the order of the conditions. In the previous study [14], TF increased after OS-1 intake by 3.7 Hz at 30 min and 5.4 Hz at 60 min, respectively. In the present study, TF increased by 6.5 ± 4.9 (immediately post-DHR) to 13.6 ± 6.0 Hz (65 min post-DHR) for the OS-1 condition (Fig. 2). It is possible that the increases in TF after OS-1 ingestion suggest a decrease in muscle cramp susceptibility. It is important to note that OS-1 contains glucose (18,000 mg/L), and serum electrolyte concentrations were not largely different from the baseline values and between conditions as shown in Table 1. Thus, it might be that the increase in TF was more due to glucose than electrolytes. Unfortunately, glucose concentration in the blood was not measured in the present study. It is necessary to add the same amount of glucose to spring water to examine the effects of glucose on muscle cramp in a future study.

Behringer et al. [31] reported that orally administered transient receptor potential vanilloid 1 (TRPV1) and ankyrin 1 (TRPA1) activators decreased muscle cramp susceptibility. It is possible that sodium in OS-1 stimulated TRP receptors in the gastrointestinal tract. It would be interesting to investigate how spring water or OS-1 intake affects oropharyngeal reflex, muscle spindles, Golgi tendon organs and alpha motor neurons, and how a small change in electrolyte concentrations in the extracellular fluid surrounding muscle fibres could affect muscle cramp susceptibility. It is also necessary to include female participants, athletes, and other age groups to confirm the present study findings. The goal of fluid intake during exercise is to prevent excessive dehydration and changes in electrolyte balance [32]. Evans et al. [33] stated that the addition of sodium to a rehydration solution is beneficial for maintenance of fluid balance due to its effect on extracellular fluid osmolality and volume. Thus, to prevent EAMC, ingesting OS-1 appears to be effective, but further research is warranted to investigate how OS-1 works to reduce the muscle cramp susceptibility. In the present study, no muscle cramp assessment was performed during exercise, and no voluntary muscle cramp was observed. It would be interesting to observe whether muscle cramp does not occur during exercise, when OS-1 or other ORS is ingested during exercise.

## Conclusion

It was concluded that spring water intake during exercise in the heat increased muscle cramp susceptibility after exercise (downhill running), and ingestion of OS-1 decreased the muscle cramp susceptibility. These were in line with the findings of our previous study [14] showing that spring water intake after dehydration made muscles more susceptible to muscle cramp, but when OS-1 was consumed, the muscle cramp susceptibility was reduced. It should be further investigated as to what and how much electrolytes should be contained in the beverage, and whether commercially available sport drinks that contain some electrolytes are as effective as OS-1 in reducing muscle cramp susceptiblilty.

## Data Availability

All data presented in the manuscriot are available upon request.

## Abbreviations

ANOVA: Analysis of variance
EAMC: Exercise-associated muscle
DHR: Downhill running
Hb: Hemoglobin
Hct: Hematocrit
HR: Heart rate
ORS: Oral rehydration solution
RPE: Rating of perceived exertion
TF: Threshold frequency
TROV1: Transient receptor potential vanilloid 1
TRPA1: Transient receptor potential ankyrin 1
SD: Standard deviation
